# Evaluation of diagnostic criteria and red flags of myelin oligodendrocyte glycoprotein encephalomyelitis in a clinical routine cohort

**DOI:** 10.1111/cns.13461

**Published:** 2020-10-13

**Authors:** Krenar Veselaj, Nicole Kamber, Myriam Briner, Christoph Friedli, Lara Diem, Kirsten Guse, Andrei Miclea, Roland Wiest, Franca Wagner, Hilary Grabe, Mathias Abegg, Michael P. Horn, Sandra Bigi, Andrew Chan, Robert Hoepner, Anke Salmen

**Affiliations:** ^1^ Department of Neurology Inselspital Bern University Hospital University of Bern Bern Switzerland; ^2^ Department of Diagnostic and Interventional Neuroradiology Inselspital Bern University Hospital University of Bern Bern Switzerland; ^3^ Department of Ophthalmology Inselspital Bern University Hospital University of Bern Bern Switzerland; ^4^ Department of Clinical Chemistry Inselspital Bern University Hospital University of Bern Bern Switzerland; ^5^ Department of Pediatrics Division of Child Neurology University Children’s Hospital Bern Inselspital Bern University Hospital University of Bern Bern Switzerland

**Keywords:** cerebrospinal fluid, multiple sclerosis, myelin oligodendrocyte glycoprotein, neuromyelitis optica spectrum disorders

## Abstract

**Aims:**

Myelin oligodendrocyte glycoprotein antibodies (MOG‐IgG) have been proposed to define “MOG encephalomyelitis” (MOG‐EM), with published diagnostic and “red flag” criteria. We aimed to evaluate these criteria in a routine clinical setting.

**Methods:**

We retrospectively analyzed patients with borderline/positive MOG‐IgG and applied the diagnostic and red flag criteria to determine likelihood of MOG‐EM diagnosis. Para‐/clinical parameters were described and analyzed with chi‐square test.

**Results:**

In total, 37 patients fulfilled MOG‐EM diagnostic criteria (female‐to‐male ratio: 1.6:1, median onset age: 28.0 years [IQR 18.5‐40.5], n = 8 with pediatric onset). In 24/37, red flags were present, predominantly MOG‐IgG at assay cutoff and/or MRI lesions suggestive of multiple sclerosis (MS). As proposed in the consensus criteria, these patients should rather be described as “possible” MOG‐EM. Of these, we classified 13 patients as “unlikely” MOG‐EM in the presence of the red flag “borderline MOG‐IgG” with negative MOG‐IgG retest *or* coincidence of ≥1 additional red flag. This group mainly consisted of patients diagnosed with MS (n = 11). Frequency of cerebrospinal fluid (CSF‐)—specific oligoclonal bands (OCB) is significantly lower in definite vs possible and unlikely MOG‐EM (*P* = .0005).

**Conclusion:**

Evaluation of diagnostic and red flag criteria, MOG‐IgG retesting (incl. change of assay), and CSF‐specific OCB are relevant in clinical routine cohorts to differentiate MOG‐EM from MS.

## Graphical abstract

Differential diagnosis of MOG encephalomyelitis vs. MS is crucial. Application of consensus diagnostic criteria and proposed red flags is helpful in a clinical routine cohort, and MOG‐IgG retesting (incl. change of assay) as well as evaluation of CSF‐specific OCB may further help in the distinction of the two entities.

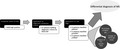

## 1 INTRODUCTION

The association of immunoglobulin G (IgG) autoantibodies directed against myelin oligodendrocyte glycoprotein (MOG) to different disease entities has been discussed^1^, ^2^: MOG‐IgG was initially described in multiple sclerosis (MS), neuromyelitis optica spectrum disorders (NMOSD), relapsing inflammatory optic neuritis (RION), acute demyelinating encephalomyelitis (ADEM), or limbic encephalitis.^3^, ^4^, ^5^, ^6^, ^7^, ^8^, ^9^ Yet, disorders associated with MOG‐IgG have recently been proposed to form their own disease entity, MOG encephalomyelitis (MOG‐EM).^2^, ^10^

Diagnostic criteria and a list of atypical conditions for MOG‐EM (“red flags”) have been suggested.^2^ However, these criteria have not yet been validated, and MOG‐EM is associated with considerable phenotypic variability among different age groups.^7^, ^9^, ^11^, ^12^, ^13^, ^14^, ^15^, ^16^

Differentiation of MOG‐EM from other inflammatory central nervous system (CNS) disorders, especially the most common one MS, is crucial as it harbors therapeutic implications. In contrast to MS, treatment (and even necessity of long‐term treatment) of MOG‐EM is thus far unclear as there are no established predictive markers to differentiate monophasic vs relapsing disease. Historically, MOG‐IgG has been described as a potential part of MS pathophysiology^6^ demonstrating histopathological similarities between MOG‐EM and MS.^17^ Differentiation between the two diseases remains challenging in routine clinical practice due to overlapping features and test‐inherent issues such as false‐positive findings.^2^, ^18^, ^19^

We retrospectively applied the proposed diagnostic criteria to a monocentric cohort of patients with borderline and positive results for MOG‐IgG as detected in clinical practice in order to critically evaluate the likelihood of MOG‐EM vs MS.

## 2 METHODS

### 2.1 Study design

In this retrospective study, clinical records of patients of the Dept. of Neurology or Child Neurology were screened for the presence of test results of MOG‐IgG obtained during routine clinical workup between January 2015 (start of assay availability) and August 2018 (data cutoff date). Clinical and paraclinical data were included of visits for a documentation period from January 2000 to August 2018, and the history of previous relapses before this documentation period was included as reported by patients during the visits. All patients with at least one positive or borderline result were included in the analyses.

We assessed demographics (age at onset, sex, comorbidities), disease characteristics (type of first symptoms, diagnosis, immunotherapies, relapses, expanded disability status scale [EDSS]^20^), laboratory parameters (MOG‐IgG [n = 37], aquaporin‐4 [AQP4‐]IgG [n = 37], ANA [n = 33], p‐ANCA/c‐ANCA [n = 33], rarely other antibodies, Table 2), cerebrospinal fluid (CSF), and MRI results. Results of CSF oligoclonal band (OCB) analysis were obtained from routine diagnostics (n = 36, isoelectric focusing, silver staining).

### 2.2 Autoantibody testing

The presence of MOG‐IgG in the serum of all patients was assessed using two different cell‐based assays: (a) until 04/2017, a FACS assay was performed at the University Hospital Basel, Prof. Derfuss, Basel, Switzerland, abbreviated as FACS. These samples were sent overnight and further processed according to the published protocol.^5^, ^21^ (b) since 05/2017, a commercial indirect immunofluorescence test using HEK 293 cells expressing either AQP‐4 or MOG (FA 1128‐1005‐1; Euroimmun AG, measured in‐house [MPH] according to the manufacturer's instructions, abbreviated as EUROIMMUN). Results of the FACS assay were classified as positive, negative, or borderline. Results of the in‐house assay EUROIMMUN were expressed as titers (<1:10 negative, 1:10 borderline, >1:10 positive). In order to assess the qualitative robustness of the two different methods, only a small number of samples (n = 6) taken at the same date were tested in both assays, n = 3 negative in both assays, n = 2 positive in both assays (Table 2, pat. 16 and 27), and n = 1 borderline in FACS, negative in EUROIMMUN (Table 2, pat. 20).

All test results are summarized for all patients in Table 2. MOG‐IgG was available for each patient from 1 up to 4 times.

All other autoantibodies (ANA, p‐ and c‐ANCA, and paraneoplastic antibodies) were measured in the clinical routine laboratory.

### 2.3 MRI

Cerebral and spinal MRI scans were performed with in‐house standard protocols for demyelinating disorders: MR images were acquired on 3 Tesla (T) and 1.5T MR scanners (Magnetom Verio 3T; Magnetom Trio 3T; Magnetom Avanto 1.5T and Magnetom 1.5T Aera; Siemens Healthcare) with the standardized MS protocol. Generally, for follow‐up scans, the same scanner was used as in the first MRI scan. All images were routinely evaluated by trained neuroradiologists. For this analysis, all scans were manually re‐evaluated by the investigators for the presence of MRI red flags as defined in Table 1.

**Table 1:** Numbering of red flags, adapted from Jarius et al.^2^ Red flags are defined as conditions that should prompt physicians to challenge a positive test result and to consider retesting the patient, ideally using an alternative, that is, methodologically different, cell‐based assay^2^

1	Chronic progressive disease course
2	Sudden onset of symptoms (<4 h)
3	Continuous worsening of symptoms over weeks
4	Lesion adjacent to a lateral ventricle that is ovoid/round or inferior temporal lobe lesion or Dawson's finger type lesions (ie, “lesions suggestive of MS”)
5	Active brain MRI with silent increase of lesion burden between relapses (limited evidence)
6	Bi‐/trispecific MRZ reaction (not available for our data)
7	MOG‐IgG at assay cutoff with/without atypical presentation
8	Positive MOG‐IgM/‐IgA with negative MOG‐IgG (not available for our data)
9	MOG‐IgG in CSF, but not in serum (not available for our data)
10	AQP4‐IgG/ MOG‐IgG double positivity (retesting recommended)
11	Findings suggesting diagnoses other than MOG‐EM, MS or NMOSD
12	Combined central and peripheral demyelination

Abbreviations: AQP4, aquaporin‐4; CSF, cerebrospinal fluid; h, hours; IgG/M/A, immunoglobulin G/M/A; MOG, myelin oligodendrocyte glycoprotein; MOG‐EM, MOG encephalomyelitis; MRI, magnetic resonance imaging; MRZ, measles/rubella/zoster; MS, multiple sclerosis; NMOSD, neuromyelitis optica spectrum disorder.

### 2.4 Application of diagnostic consensus criteria for MOG‐EM and “red flags”

The recently published criteria and proposed “red flags”^2^ were evaluated for the initial cohort of n = 40 patients. The red flags “bi‐/trispecific MRZ reaction,” “MOG‐IgM/‐IgA with negative MOG‐IgG,” and “MOG‐IgG in CSF, but not in serum” could not be evaluated as these are not part of the routine clinical workup in our center (Table 1).

For patients fulfilling the diagnostic criteria, we assessed the presence of red flags and thus defined three groups:

- Definite MOG‐EM (as proposed by^2^)
- Possible MOG‐EM (as proposed by^2^)
- Unlikely MOG‐EM: presence of the red flag “MOG‐IgG at assay cutoff” *plus* a negative retest for MOG‐IgG *or* at least one additional red flag.

### 2.5 Analysis and statistics

Descriptive statistics were used to summarize patient data. For categorical variables, absolute and relative frequencies are reported. For continuous variables, median and interquartile range (IQR), if deemed informative, also minimum (min.) and maximum (max.) are given. Frequencies of missing data are disclosed for each parameter. Group comparisons were performed with Kruskal‐Wallis test as a nonparametric test. Chi‐square test was used to calculate difference in OCB and clinical diagnosis distribution between MOG‐EM groups. Statistical significance level was set to *P* < .05.

All analyses were performed with GraphPad Prism version 7.03 for Windows, GraphPad Software.

### 2.6 Ethics approval

This study was approved by the responsible ethics committee (cantonal ethics committee Bern, registration no. KEK‐BE 2017‐01369). For this retrospective analysis, pseudonymized patient data were included. A separate informed consent was waived by the committee. For patients seen after the introduction of the general consent (Feb‐2015), the presence of the patients’ general consent was checked before inclusion in the analysis. Pediatric patients and their legal representatives were individually asked for their consent to the pseudonymized use of data by their treating physician (SB), as these are not covered by the general consent.

## 3 RESULTS

### 3.1 Characteristics of the cohort

We identified n = 40 patients with borderline or positive MOG‐IgG. Of this initial group, n = 3 patients did not demonstrate clinical findings compatible with a demyelinating event (functional disorder, episodic vertigo, depression, each n = 1). Two of these three had normal brain MRI scans and were tested positive in FACS with no retest available and a negative retest in EUROIMMUN, respectively. For the third patient tested borderline in EUROIMMUN, neither a retest, nor further clinical or paraclinical data were available. These three presumably false‐positive/borderline patients *not* fulfilling the diagnostic criteria were excluded from further analyses.

The remaining 37 patients fulfilling the diagnostic criteria were included in the analyses.

**Figure 1:**
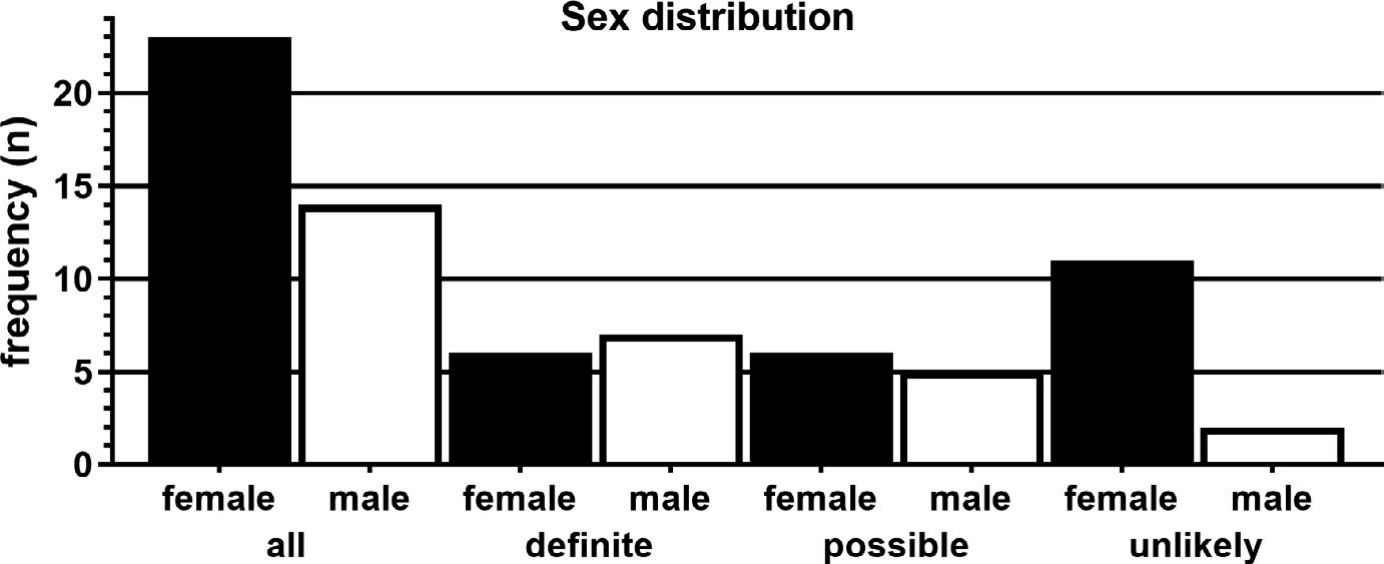
Sex distribution of the cohort. Female (black bars) and male (white bars) patients according to the groups: whole cohort (“all,” n = 37), “definite” (n = 13), “possible” (n = 11), and “unlikely” (n = 13) MOG encephalomyelitis

The female‐to‐male ratio was 1.6:1 (Figure 1). The median age at onset was 28.0 years (IQR 18.5‐40.5, min. 9.0, max. 66.0). The last documented median EDSS was 2.0 (IQR 1.5‐3.0, min. 0, max. 8.0).

**Figure 2:**
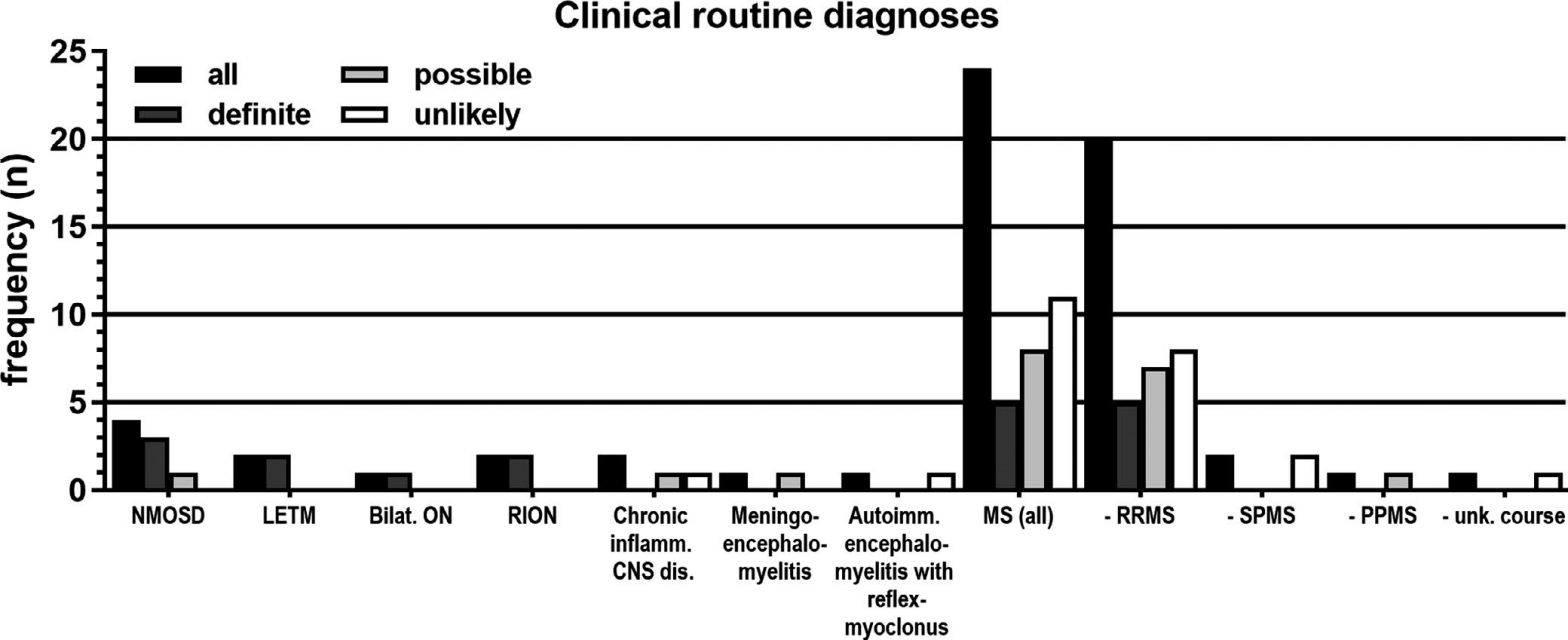
Diagnoses as given in clinical routine (before establishment of the term “MOG encephalomyelitis” (MOG‐EM) and respective definition of diagnostic criteria). Black bars: whole cohort (n = 37), dark gray bars: “definite” MOG‐EM (n = 13), light gray bars: “possible” MOG‐EM (n = 11), white bars: “unlikely” MOG‐EM (n = 13). For MS, the disease course is given, additionally. Autoimm., autoimmune; Bilat., bilateral; dis., disorder; inflamm., inflammatory; LETM, longitudinal extensive transverse myelitis; NMOSD, neuromyelitis optica spectrum disorder; ON, optic neuritis; PPMS, primary progressive MS; RION, relapsing inflammatory optic neuritis; RRMS, relapsing‐remitting MS; SPMS, secondary progressive MS; unk., unknown

As part of routine clinical care, patients were given different diagnoses of demyelinating CNS disorders (Figure 2).

**Figure 3:**
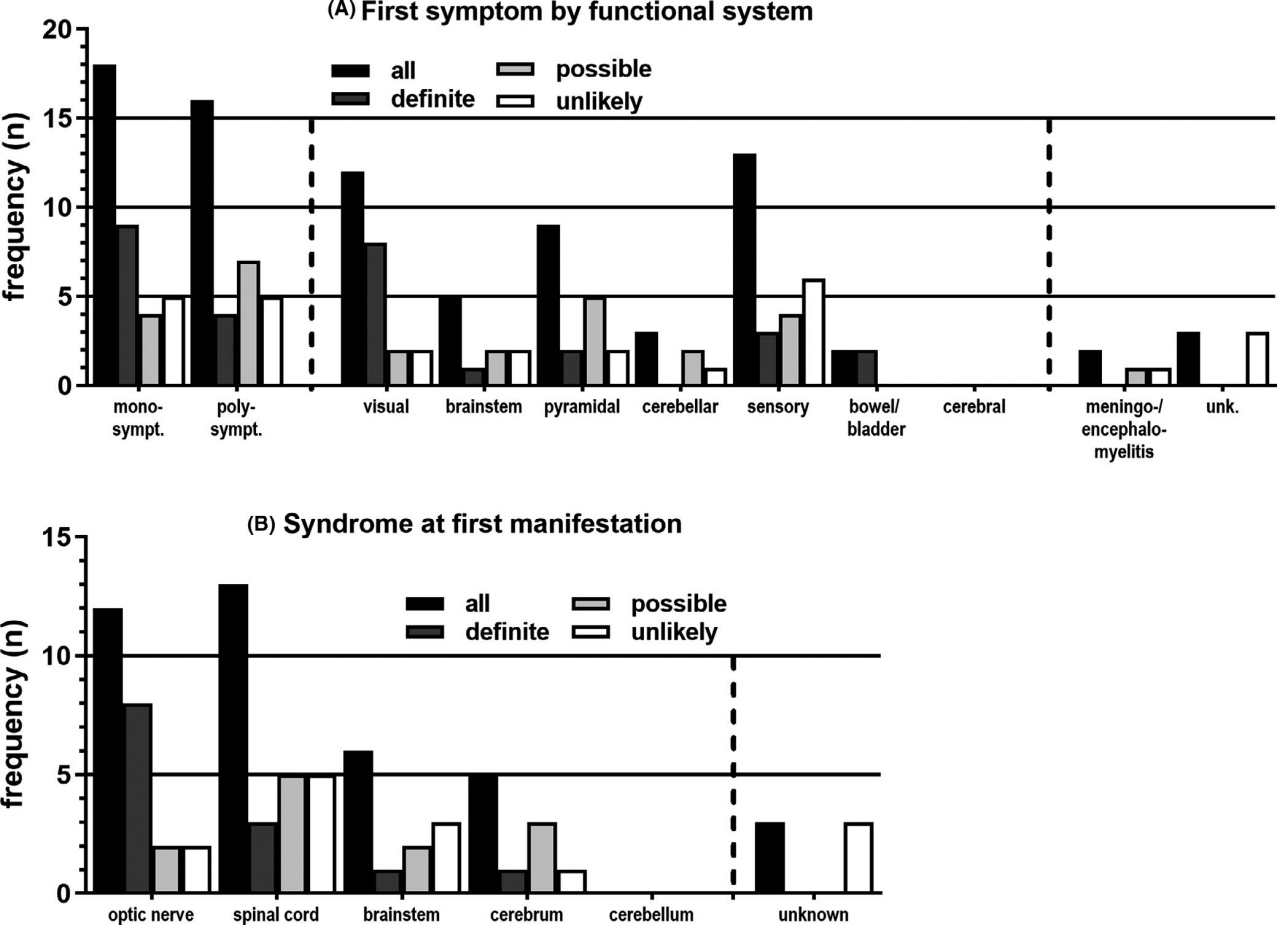
A, First symptom at disease onset, classified by EDSS functional system scores. If more than 1 symptom was reported, patients were counted as “polysymptomatic”, otherwise “monosymptomatic.” Black bars: whole cohort (n = 37), dark gray bars: “definite” MOG‐EM (n = 13), light gray bars: “possible” MOG‐EM (n = 11), white bars: “unlikely” MOG‐EM (n = 13). For two patients, classification by functional system score was not deemed appropriate (multifocal, meningo‐/encephalomyelitis); for three patients, symptoms at onset were unknown. Monosympt., monosymptomatic; polysympt., polysymptomatic; unk., unknown. B, Syndrome at first manifestation. Black bars: whole cohort (n = 37), dark gray bars: “definite” MOG‐EM (n = 13), light gray bars: “possible” MOG‐EM (n = 11), white bars: “unlikely” MOG‐EM (n = 13). For three patients, unknown syndrome at onset

Classified by EDSS functional system scores, symptoms at onset were almost equally poly (n = 16)‐ or monosymptomatic (n = 18) with visual (n = 12), sensory (n = 13), and pyramidal (n = 9) being the most frequent (Figure 3A).

The syndrome at first manifestation was mainly spinal cord or optic nerve involvement (Figure 3B).

### 3.2 Evaluation of “red flags” and classification of likelihood of MOG‐EM

A large proportion of patients (24/37) demonstrated at least one red flag (Table 2) and should thus be defined as “possible” MOG‐EM.^2^ Most frequently, the MRI red flag “lesions suggestive of MS” was present (n = 18/37). In 16/37 patients, MOG‐IgG was detected at the assay cutoff. Progressive disease course was described in 5/37 patients. The MRI red flag “silent increase in lesion burden” was noted for 1/37 patient.

**Table 2:** Characteristics of the cohort and red flags per patient (as numbered in Table 1) with classification of patients in MOG‐EM categories “definite,” “possible,” and “unlikely”

Patient number	Age at onset (years)	Sex	Last documented EDSS	Red flags	Likelihood classification	Details on MOG‐IgG testing	OCB pattern	Other autoantibodies	Other autoimmune conditions	Para‐/postinfectious onset
1	40	Female	6.5	1,4,7	Unlikely	FACS: borderline, no retest available	2	No	Autoimmune hepatitis	
2	58	Female	5.5	1,4,5	Possible	EUROIMMUN: positive, no retest available	2	ANA 1:1280	Undifferentiated connective tissue disease	
3	22	Male	1.5	—	Definite	tested thrice, FACS: positive	2	No	No	
4	21	Female	n.a.	7	Possible	FACS: borderline, no retest available	2	ANA 1:160	No	Unknown pathogen
5	21	Male	2.5	7	Possible	FACS: borderline, no retest available	2	No	No	
6	32	Male	6.0	4,7	Possible	1st test FACS, 2nd test EUROIMMUN: confirmed borderline	1	No	No	
7	66	Male	2.0	4,7	Unlikely	1st test FACS: borderline 2nd test EUROIMMUN: negative	2	ANA 1:320	No	
8	25	Male	4.0	4	Possible	Tested thrice, FACS and EUROIMMUN: positive	n.a.	No	No	
9	55	Male	1.5	4	Possible	FACS: positive, no retest available	2	ANA 1:320, p‐ANCA 1:160	No	
10	35	Female	0	4,7	Unlikely	FACS: borderline, no retest available	2	No	No	
11	25	Female	1.0	4,7	Unlikely	1st test FACS: borderline, 2nd test EUROIMMUN: negative	2	ANA 1:160	No	
12	28	Female	3.5	4,7	Unlikely	1st test FACS: borderline, no retest available	2	n.a.	No	
13	19	Female	2.0	4	Possible	FACS: positive, no retest available	2	No	No	
14	30	Female	2.0	7	Unlikely	1st test EUROIMMUN: borderline, 2nd test EUROIMMUN: negative	2	No	No	
15	34	Male	1.5	—	Definite	FACS: positive, no retest available	2	No	No	
16	17	Female	2.0	—	Definite	Tested 4 times, FACS and EUROIMMUN: positive	1	No	No	
17	52	Male	1.0	—	Definite	FACS: positive, no retest available	1	No	No	
18	31	Male	1.5	—	Definite	Tested twice, FACS and EUROIMMUN: positive	1	No	No	
19	18	Female	8.0	1,4,7	Unlikely	1st test FACS: borderline, 2nd test EUROIMMUN: negative	2	No	No	
20	41	Female	1.5	7	Unlikely	1st and 2nd test FACS: borderline, 3rd test EUROIMMUN: negative	2	No	No	
21	53	Female	1.0	7	Possible	FACS: borderline, no retest available	1	No	No	
22	21	Female	n.a.	4	Possible	FACS: positive, no retest available	2	No	No	
23	15	Female	n.a.	‐	Definite	Tested 4 times 1st and 2nd test FACS: positive, 3rd test EUROIMMUN: negative, 4th test EUROIMMUN: positive	1	No	No	
24	16	Female	2.5	4,7	Unlikely	1st test FACS: borderline, 2nd test EUROIMMUN: negative	2	n.a.	No	
25	29	Male	n.a.	1,4,7	Unlikely	EUROIMMUN: borderline, no retest available	4	SOX‐1 borderline	No	
26	17	Female	2.5	4	Unlikely	1st test FACS: negative, 2nd test FACS: positive, 3rd test EUROIMMUN: negative	2	No	Chronic juvenile arthritis	
27	30	Female	2.0	—	Definite	Tested thrice, FACS and EUROIMMUN: positive	1	No	No	
28	17	Male	1.5	—	Definite	Tested 4 times FACS and EUROIMMUN: positive	1	No	No	
29	28	Female	2.5	4,7	Unlikely	EUROIMMUN: borderline, no retest available	2	No	No	
30	45	Male	3.0	1	Possible	FACS: positive, no retest available	2	No	No	
31	21	Female	1.0	—	Definite	Tested thrice, 1st test FACS: positive, 2nd test EUROIMMUN: positive, 3rd test EUROIMMUN: negative	2	No	No	
32	55	Female	1.5	4	Possible	FACS: positive, no retest available	2	No	No	
33	11	Female	n.a.	—	Definite	tested twice, EUROIMMUN: 1st positive, 2nd borderline	4	n.a.	No	VZV
34	9	Female	n.a.	—	definite	tested thrice, EUROIMMUN: 1st positive, 2nd and 3rd negative	1	No	No	
35	14	Female	2.0	4,7	Unlikely	EUROIMMUN: borderline, no retest available	2	n.a.	No	
36	24	Male	8.0	—	Definite	Tested 5 times, EUROIMMUN: 1st positive, 2nd‐4th negative, 5th borderline	4	No	No	Unknown pathogen
37	45	Male	3.0	—	Definite	tested thrice, EUROIMMUN: 1st borderline, 2nd and 3rd positive post hoc test of serum before disease onset during EHEC sepsis, EUROIMMUN: negative	1	No	No	EHEC

Abbreviations: ANA, antinuclear antibodies; EDSS, expanded disability status scale; EHEC, enterohemorrhagic Escherichia coli; EUROIMMUN, commercial assay, used in‐house; FACS, initial assay (Basel); IgG, immunoglobulin G; MOG, myelin oligodendrocyte glycoprotein; n.a., not available; OCB patterns: 1—type 1 (polyclonal), 2—type 2 (CSF‐specific OCB), 4—type 4 (identical OCB in CSF and serum), SOX‐1—SRY‐box transcription factor 1, VZV—varicella zoster virus; OCB, oligoclonal bands; p‐ANCA, perinuclear antineutrophil cytoplasmic antibodies.

With the classification described above, that is, adding the category “unlikely,” patients were categorized as “definite” (n = 13), “possible” (n = 11), and “unlikely” (n = 13) MOG‐EM.

We classified one patient (pat. 26, Table 2) as “unlikely” with a single positive test result (FACS), but a negative pretest (FACS) and post‐test (EUROIMMUN) for MOG‐IgG and the additional red flag “lesions suggestive of MS.” This single patient is formally not consistent with the definitions given above as the intermittent FACS result was indicated as positive, not borderline.

Among the “possible” group is one patient with confirmed borderline results in the two different assays (FACS and EUROIMMUN) and the red flag “lesions suggestive of MS” (Table 2, pat. 6). Interestingly, this patient did not demonstrate CSF‐specific OCB and experienced ongoing clinical and MRI disease activity while treated with glatiramer acetate, beta‐interferon, dimethyl fumarate, and natalizumab, but is stable since introduction of B‐cell depletion (15 months of follow‐up since initiation of rituximab).

Re‐evaluating the cohort characteristics per diagnosis likelihood groups, the female‐to‐male ratio shifts for “definite” and “possible” MOG‐EM close to 1:1 (Figure 1). Age at onset is not significantly different between groups (“definite”: median 22.0 years [IQR 16.0‐32.5, min. 9.0, max. 52.0], “possible”: median 32.0 years [IQR 21.0‐55.0, min. 19.0, max. 58.0], “unlikely”: median 28.0 years [IQR 17.5‐37.5, min. 14.0, max. 66.0], *P* = .18), neither is the last documented EDSS (“definite” [n = 10]: median 1.5 [IQR 1.4‐2.3], “possible” [n = 9]: median 2.5 [IQR 1.5‐4.8], “unlikely” [n = 12]: median 2.3 [IQR 1.6‐3.3], *P* = .38).

Regarding clinical routine diagnoses, “unlikely” and “possible” MOG‐EM mainly consisted of patients diagnosed with MS (11 of 13 and 8 of 11, respectively, vs 5 of 13 in “definite” MOG‐EM, *P* = .04, Figure 2).

For classification by EDSS functional system scores (Figure 3A) and by syndrome (Figure 3B) at onset, “definite” MOG‐EM seems prone to involvement of the optic nerve (8 of 13) in our cohort. Due to the various groups of small size, no formal statistical analysis was run over this part.

### 3.3 Pediatric‐onset cases

All eight pediatric‐onset patients demonstrated a spinal cord or optic nerve syndrome. The two youngest children in our cohort (11 and 9 years) experienced initial high‐titer MOG‐IgG (1:160 and 1:320) that went back to borderline within 2 months and negative within 4 months, respectively, with thus far monophasic disease course (follow‐up of 10 months after onset for both patients). Patients aged 17, 15, and 17 years (pat. 16, 23, and 28, Table 2) all experienced recurrent disease attacks (n = 2 RION, n = 1 with first attack of a bilateral ON, second/third attack: encephalitis and LETM in short sequence). These pediatric‐onset patients were classified as “definite” MOG‐EM (n = 5). The remaining n = 3 patients (aged 16, 17, 14 years) were classified as “unlikely” MOG‐EM with the diagnosis of relapsing‐remitting MS (RRMS, pat. 24, 26, 35, Table 2). No ADEM manifestations were present in our incidental cohort.

### 3.4 Coexisting autoantibodies and autoimmunity

None of the tested sera showed positivity for coexisting AQP4‐IgG (0/37). A positive ANA titer was detected in 5/33 patients (Table 2). Patients with positive ANA were divided into “unlikely” (n = 2) and “possible” (n = 3) MOG‐EM, respectively.

A patient with an atypical clinical manifestation (classified as autoimmune encephalomyelitis with reflex‐myoclonus, classified “unlikely” MOG‐EM) was extensively re‐evaluated at the age of 67 years. A MOG‐IgG titer of 1:10 was detected, combined with a low‐titer SOX‐1 antibody. A large panel of other autoantibodies in this patient was negative, including GAD and IA‐1 antibodies (coexisting Diabetes mellitus). Due to repeated treatment with intravenous immunoglobulins after this first testing, retesting was not performed. This male patient experienced first symptoms at the age of 29 years. A neoplastic comorbidity was not found.

Secondary autoimmune diagnoses were found in three patients (autoimmune hepatitis, undifferentiated connective tissue disease, chronic juvenile arthritis, each n = 1, Table 2). These patients were divided into “unlikely” (n = 2) and “possible” (n = 1) MOG‐EM.

### 3.5 Para‐/postinfectious disease onset

In our cohort, n = 4 patients demonstrated a para‐ or postinfectious disease onset (Table 2, “definite” [n = 3] and “possible” [n = 1] MOG‐EM). In two patients (aged 21 years, female and 24 years, male), no pathogen was detected in an extensive workup. Diagnoses were acute meningoencephalomyelitis, presenting with meningismus, phono‐/photophobia, and fever (39°C) and longitudinal extensive transverse myelitis (LETM), presenting 1 week after onset of pharyngitis/tonsillitis with persisting fever (38°C) at the day of admission under antibiotic treatment (cefuroxime), respectively.

In a female pediatric patient (aged 11 years) with LETM, a VZV primoinfection was detected, simultaneously.

A male patient (aged 45 years) experienced a severe EHEC sepsis with multiorgan failure. With a first manifestation of optic neuritis (ON, right) 7 weeks later and sequential recurrent and bilateral ON, RION was diagnosed. MOG‐IgG was first tested approximately 12 weeks after EHEC sepsis (titer 1:10, during second attack) and meanwhile confirmed thrice with increasing titers (1:20; 1:40; 1:40). Serum available from the acute EHEC sepsis phase was retrospectively tested and negative for MOG‐IgG.

### 3.6 Evaluation of CSF‐specific oligoclonal bands in MOG‐EM likelihood groups

Results of OCB testing were available for 36 patients (Table 2). Altogether, type 2 OCB (CSF‐specific) were documented in 23 patients of the whole cohort, type 1 OCB (polyclonal) in 10 and type 4 OCB (identical bands in CSF and serum) in 3 patients, respectively. As described before, only type 2 OCB (and type 3 which did not occur in our cohort) are CSF‐specific, whereas type 1 and type 4 OCB do not represent local IgG synthesis.^2^, ^22^

The frequency distribution of the presence of CSF‐specific OCB vs absence of CSF‐specific OCB is significantly different between the groups “definite,” “possible,” and “unlikely” (*P* = .0005, Table 3).

**Table 3:** Frequency distribution of presence vs absence of cerebrospinal fluid (CSF‐)‐specific oligoclonal bands (OCB) of the patients according to MOG encephalomyelitis (MOG‐EM) groups “definite,” “possible,” and “unlikely” (*P* = .0005, chi‐square test)

	Definite MOG‐EM	Possible MOG‐EM	Unlikely MOG‐EM
CSF‐specific OCB	3	8	12
No CSF‐specific OCB	10	2	1

Abbreviations: CSF, cerebrospinal fluid; MOG‐EM, MOG encephalomyelitis; OCB, oligoclonal bands.

## 4 CONCLUSION

MOG encephalomyelitis has recently been proposed to form its own disease entity.^1^, ^2^, ^10^ However, both the clinical spectrum and differential diagnosis are thus far not well determined and proposed diagnostic criteria need broader validation which is challenging in a rare condition. In clinical practice, different methods of MOG‐IgG testing harbor relevant differences in sensitivity and specificity.^23^ Occurrence of low‐titer MOG‐IgG in patients with MS may cause clinically relevant delays in therapeutic decision making and ultimately represent false‐positive findings.^18^

We present a monocentric cohort of 37 well‐characterized patients with suspected MOG‐IgG‐associated disorder and retrospectively applied the proposed diagnostic criteria and red flags as well as OCB to differentiate MOG‐EM from MS.

Regarding basic demographic and clinical results, our cohort is in line with existing data.^14^, ^15^, ^24^ The shift of the female‐to‐male ratio to 1:1 after exclusion of “unlikely” MOG‐EM might rather be related to sample size.

We retrospectively applied the proposed diagnostic criteria and red flags^2^ to our cohort of suspected MOG‐EM. A remarkable proportion of patients had either MOG‐IgG at the assay cutoff and/or MRI lesions suggestive of MS. This prompted us to define a group of “unlikely” MOG‐EM in addition to the “possible” group as suggested in the consensus paper.^2^

With this classification, significantly more patients with a clinical diagnosis of MS prior to antibody testing were grouped as “unlikely” or “possible” than “definite”.

It is thus important to develop tools that help to better differentiate MOG‐EM from MS, in particular as this harbors therapeutic implications: MS can be treated with several immunotherapies, but MOG‐EM may not only not respond, but even worsen under established MS treatments.^13^, ^14^

Borderline MOG‐IgG titers with a negative retest or additional MRI red flags may thus lead us to reject the diagnosis of MOG‐EM. However, given our small cohort, corroboration of our findings is necessary.

It has to be kept in mind that assay‐related issues, that is, false‐positive findings, may contribute to our findings primarily with regard to the commercially available EUROIMMUN assay—as putatively in many other centers. Whereas the EUROIMMUN assay performs less well than live cell‐based assays in terms of the positive predictive value, it still generally provides good agreement with the latter with comparable specificity and negative predictive values.^23^ As a limitation, we cannot directly compare EUROIMMUN results to the performance of the previous FACS assay as only very few samples of the same date were simultaneously analyzed in both assays. A direct comparison between the assays was thus not distinctly performed. For some of our patients, negative retests of borderline FACS results were detected with the EUROIMMUN assay. However, for several patients we cannot provide retest data (both for FACS and EUROIMMUN) and we do not have confirmatory results from a live cell‐based assay. As a consequence, in clinical practice, retesting should be considered in borderline results and be performed, whenever possible, using both the routinely available assay and also an additional method, preferentially a live cell‐based assay in a reference center.

Pediatric disease onset is mainly associated with optic nerve and spinal cord involvement as first symptom in our and in other cohorts.^25^, ^26^ However, as this does not sufficiently distinguish MOG‐EM from MS in children and adolescents, the differentiation toward MS is crucial as we identified 3 patients as “unlikely” MOG‐EM with our definition that were all diagnosed with RRMS and experienced sequential relapses. Corroborating previous findings, these 3 patients were older at onset (16, 17, and 14 years, respectively) and demonstrated OCB type 2 (Table 2).^27^ This is therapeutically relevant as younger patients with “true” transient MOG‐IgG are more likely to be monophasic as described in our cohort, whereas both RRMS and older adolescent patients with persisting MOG‐IgG are at risk for sequential relapses with potential severe disability and thus require immunotherapy.^1^, ^27^

Single cases of para‐/postinfectious disease manifestations of MOG‐EM were described before, both in adults and in children.^14^, ^28^ In all our patients, signs of systemic infection were still present at the onset of MOG‐EM and/or a distinct pathogen was identified. Postinfectious disease onset is likely to be underdiagnosed, especially if MOG‐EM occurs within weeks after a common trivial infection. With the negative MOG‐IgG result of the patient during EHEC sepsis, disease manifestation of MOG‐EM with RION phenotype 7 weeks later and sequential confirmed positive MOG‐IgG, our work may imply a possible underlying pathogenesis that involves triggering a general immune response^29^ or molecular mimicry processes.^30^

After classification of MOG‐EM likelihood groups, patients with coexisting ANA autoantibodies or another autoimmune disease were not present in the “definite” MOG‐EM group. As in other cohorts, the small sample size limits wide interpretations. Yet, both ANA and coexistence of other autoimmune conditions have been described to be a potential help in differentiation vs AQP4‐IgG positive NMOSD.^31^ As we did not compare to an NMOSD cohort, we cannot validate this finding with our data. Detectable (mainly low‐titer) autoantibodies including ANA and MOG‐IgG may occur within an autoimmune condition that does not qualify for MOG‐EM and even in healthy persons; these may thus be unspecific or false‐positive findings^32^, ^33^ and argue for testing of MOG‐IgG only in suspected cases of a demyelinating CNS disorder.^2^

In addition to the application of the proposed red flags, we evaluated CSF‐specific OCB. As addressed in the diagnostic criteria, the absence of CSF‐specific OCB in patients with suspected MS should trigger MOG‐IgG testing.^2^ In turn, their presence may also be used to challenge the diagnosis of MOG‐EM, especially if they occur in combination with the named red flags. This is supported by our and other data.^31^

Due to the retrospective nature of our analysis without available retesting of MOG‐IgG for all, particularly borderline‐tested, patients, we cannot exclude that false‐positive findings of MOG‐IgG are present in the “possible,” and maybe even in the “definite” group. This situation will frequently occur in clinical practice. In addition, our study has the limitation of a small sample size. Nevertheless, for a rare and recently defined condition, our cohort, albeit small, contributes to a better understanding of MOG‐EM and differential diagnoses, especially MS, corroborating known data from other small cohorts and adding new insights. The inclusion of both the proposed combination of red flags and CSF findings to challenge or even rule out the diagnosis of MOG‐EM in suspicious cases may be valuable if further confirmed in other cohorts in order to prevent treatment delays. Collaborative prospective studies are warranted to provide sufficient sample sizes and further elaboration of the differential diagnostic algorithm.

## CONFLICT OF INTEREST

K Veselaj, C Friedli, A Miclea, R Wiest, H Grabe, M Abegg, and M P Horn report no disclosures. N Kamber received travel grants from Biogen, Merck, Genzyme, and Roche, not related to this work. M Briner received travel grants from Merck and Biogen, not related to this work. L F Diem received travel grants from Merck, Biogen, Roche, and Bayer Schweiz, not related to this work. K Guse is a former employee of Biogen and current employee of CSL Behring, not related to this work. F Wagner received a research grant from the Swiss MS Society, not related to this work. S Bigi received speaker honoraria and consultancy fees from Novartis and Sanofi, not related to this work. A Chan received personal compensation for activities with Bayer, Biogen, Genzyme, Merck, Novartis, Roche, and Teva. He received research support from the Swiss National Fonds (SNF, No. 310030_172952), Genzyme, and UCB, not related to this work. R Hoepner received research and travel grants from Novartis and Biogen Idec, speaker's honoraria from Biogen, Novartis, Merck, and Almirall, and research support by the Swiss MS Society, not related to this work. A Salmen received speaker honoraria and/or travel compensation for activities with Almirall Hermal GmbH, Biogen, Merck, Novartis, Roche, and Sanofi Genzyme, and research support by the Swiss MS Society, not related to this work.

## Data Availability

Data used in preparation of the figures and tables might be shared in anonymized format on request of a qualified investigator to the corresponding author for purposes of replicating procedures and results.
